# Case Report: Dominant deafness-onychodystrophy syndrome and hypokalemic periodic paralysis in a single patient: a rare syndromic overlap

**DOI:** 10.3389/fped.2025.1674481

**Published:** 2026-02-16

**Authors:** Dong-Lan Luo, Rui-Ping Liu, Xin-Yi Hou, Zhi Liu, Bin Lu

**Affiliations:** 1 Shenzhen Maternity and Child Healthcare Hospital, Women and Children's Medical Center, Southern Medical University, Shenzhen, Guangdong Province, China; 2 Clinical Medical College, Jining Medical University, Jining, China; 3 Department of Dermatology, Affiliated Hospital of Jining Medical University, Jining, China; 4 Department of Dermatology, The Affiliated Hospital of Guizhou Medical University, Guiyang, China

**Keywords:** dominant deafness-onychodystrophy syndrome, hypokalemic periodic paralysis, *ATP6V1B2*, *CACNA1S*, deafness, channelopathies

## Abstract

Dominant deafness-onychodystrophy syndrome (DDOD) and hypokalemic periodic paralysis (HypoPP) are distinct autosomal dominant disorders caused by mutations in *ATP6V1B2* and *CACNA1S*, respectively. We describe an eight-year-old female with congenital deafness, nail dysplasia, enamel hypoplasia, and recurrent episodes of hypokalemia-induced muscle weakness. Whole-exome sequencing (WES) revealed a *de novo ATP6V1B2* nonsense variant (c.1516C>T, p.Arg506Ter) and a maternally inherited *CACNA1S* missense variant (c.1583G>A, p.Arg528His). The proband's mother and maternal grandfather carried the same *CACNA1S* variant with milder periodic weakness, indicating intrafamilial variability and reduced penetrance. This dual diagnosis broadens the phenotypic spectrum of both DDOD and HypoPP and illustrates how comprehensive genomic testing can elucidate complex, blended phenotypes. Although no direct mechanistic link between *ATP6V1B2* and *CACNA1S* has been demonstrated, their coexistence highlights the importance of considering multilocus genetic etiologies in rare diseases and supports precision medicine approaches integrating genomic diagnostics and individualized management.

## 1 Introduction

Dominant deafness-onychodystrophy syndrome (DDOD) is characterized mainly by congenital sensorineural hearing loss accompanied by dystrophic or absent nails (1). DDOD syndrome is caused by a truncating variant in *ATP6V1B2* (MIM 606939). This variant creates a premature stop codon removing five amino acids and results in a truncated protein, which has been shown to impair lysosome acidification (1–3).

Hypokalemic periodic paralysis (HypoPP) is an autosomal dominant channelopathy marked by episodic muscle weakness and hypokalemia, often triggered by rest following exertion or high-carbohydrate meals (4). Most cases are due to mutations in the *CACNA1S* (MIM 114208) gene, which encodes the alpha−1 subunit of the L-type calcium channel in skeletal muscle, with p.Arg528His being among the most common pathogenic variants (5).

Both DDOD and HypoPP are rare Mendelian disorders with distinct molecular mechanisms and clinical manifestations. However, the coexistence of two independent monogenic diseases in a single patient provides a unique opportunity to explore potential convergent pathways, and the diagnostic power of genomic sequencing in complex phenotypes. Dual or multilocus diagnoses are increasingly recognized with the widespread use of next-generation sequencing, revealing that up to 5% of individuals with suspected Mendelian disorders harbor more than one pathogenic variant (6, 7). Such cases are of high clinical relevance because they often present blended or atypical phenotypes that challenge traditional single-gene diagnostic frameworks.

Here, we report a pediatric patient presenting with congenital deafness, ectodermal abnormalities, and periodic hypokalemia. Comprehensive genetic evaluation identified pathogenic variants in both *ATP6V1B2* and *CACNA1S*, leading to a dual diagnosis of DDOD and HypoPP.

## 2 Case report

We present this case to highlight the diagnostic and mechanistic insights gained from identifying dual genetic etiologies in a single patient.

The proband was an eight-year-old girl who was born at term following an uneventful pregnancy conceived through conventional *in vitro* fertilization (IVF) without preimplantation genetic testing. At birth, she exhibited hypoplastic fingernails on the second to fourth digits of both hands and complete absence of toenails. Hearing screening at four months revealed profound bilateral sensorineural hearing loss without evidence of seizures or abnormal EEG activity. Dental examination revealed enamel hypoplasia and a high-arched palate.

At the age of five, the proband began to experience recurrent episodes of muscle weakness primarily involving the limbs, frequently accompanied by non-projectile vomiting. The vomiting consisted of gastric contents without the presence of coffee-ground material or bile. There was no associated diarrhea or fever, and bowel movements remained normal, although urinary output was decreased during attacks. The episodes typically occurred after meals and lasted from several hours to an entire day. Initially, these episodes occurred about once a month, increasing to once a week by age eight. During multiple acute episodes, laboratory testing revealed hypokalemia with serum potassium levels below the normal range, and electrocardiography demonstrated U waves consistent with the manifestations of hypokalemia. Other biochemical parameters, including renal, hepatic, and thyroid function, as well as serum calcium and magnesium, were within normal limits. She was referred for genetic evaluation due to the combination of ectodermal abnormalities, hearing loss, and periodic hypokalemia.

WES was performed to investigate the constellation of congenital sensorineural hearing loss, ectodermal abnormalities, and recurrent episodes of hypokalemia. Genomic DNA was extracted from peripheral blood and sequenced using the Illumina HiSeq platform with a mean coverage of >100×. Variants were prioritized based on clinical relevance, allele frequency in population databases (gnomAD, 1000 Genomes), and pathogenicity predictions. The analysis identified a heterozygous nonsense variant in *ATP6V1B2* (c.1516C>T, p.Arg506Ter), previously associated with dominant deafness–onychodystrophy syndrome (DDOD), and a heterozygous missense variant in *CACNA1S* (c.1583G>A, p.Arg528His), a known pathogenic mutation in HypoPP. Segregation analysis confirmed the *CACNA1S* variant was maternally inherited and co-segregated with HypoPP in the family, while the *ATP6V1B2* variant arose *de novo*. Her mother and maternal grandfather also reported similar symptoms. The clinical presentation showed notable intrafamilial variability. The grandfather described a history of mild, intermittent muscle weakness commencing in adolescence, with an estimated frequency of three episodes per month. According to family report, the frequency of these attacks diminished to approximately once per month after he reached 40 years of age, which aligns with the known natural history of HypoPP (5). In contrast, the proband's mother exhibited a much milder phenotype, with rare episodes of weakness occurring every few months and decreasing to only once every six months in recent years. This pattern is suggestive of the reduced penetrance and expressivity often observed in female carriers of *CACNA1S* variants (5, 8). Neither affected relative has hearing loss or nail abnormalities.

**Figure 1:**
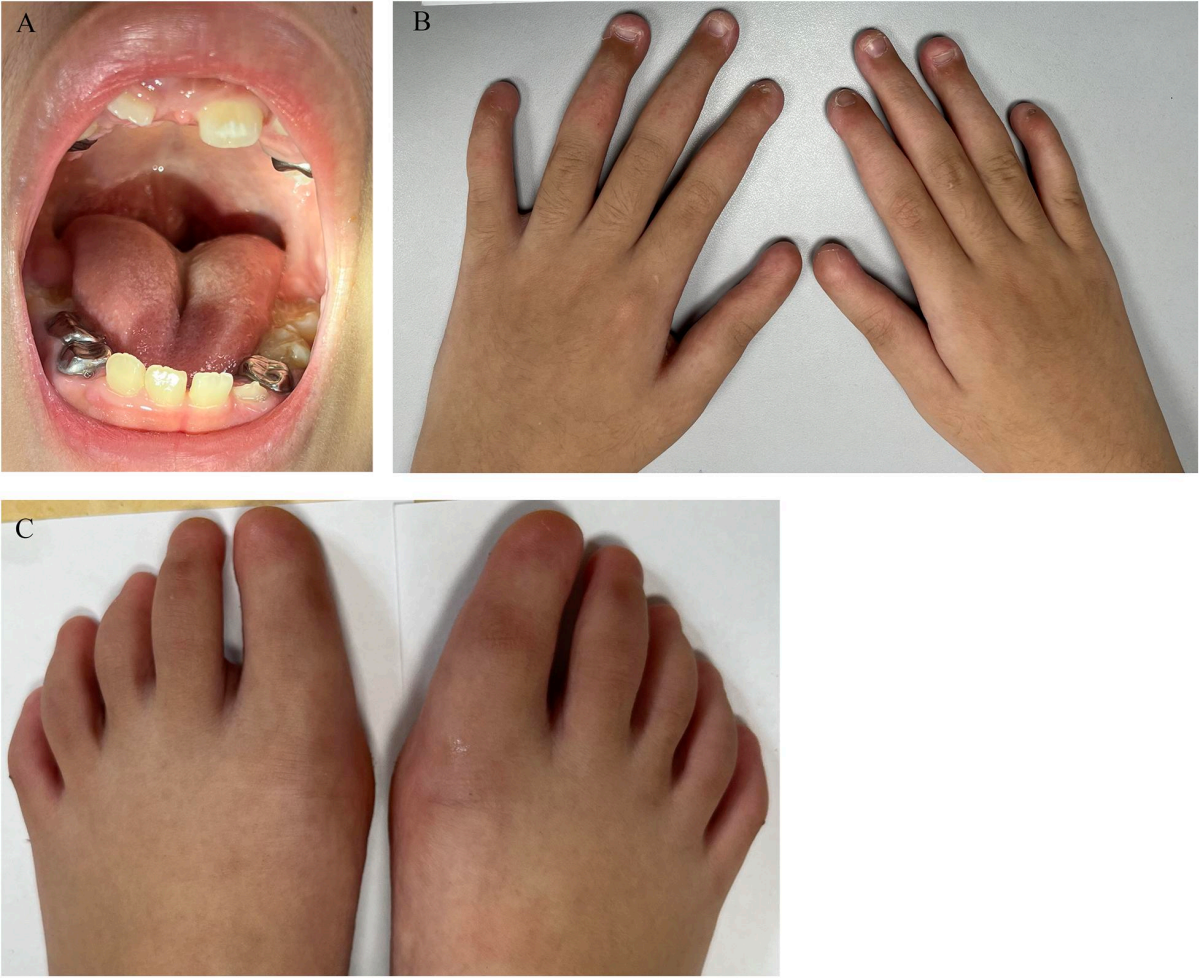
Clinical photograph of the patient. **(A)** Shows high arched palate, hypoplastic teeth. **(B)** Shows aplastic/hypoplastic fingernails. **(C)** Shows absent of all toenails.

**Figure 2:**
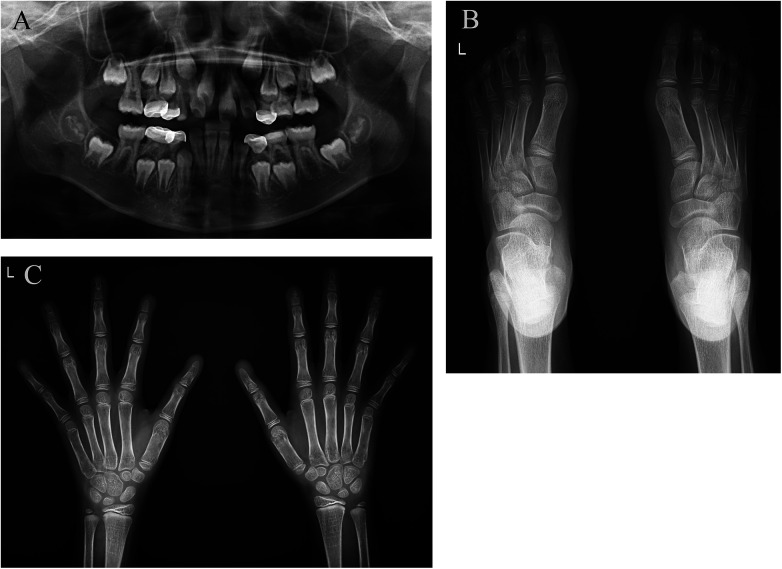
x-ray examination of the patient's teeth, hands, and feet. **(A)** Shows dental hypoplasia. **(B and C)** Show that the distal phalanx of both hands and the distal phalanx of both feet become sharp and develop deformities.

**Figure 3:**
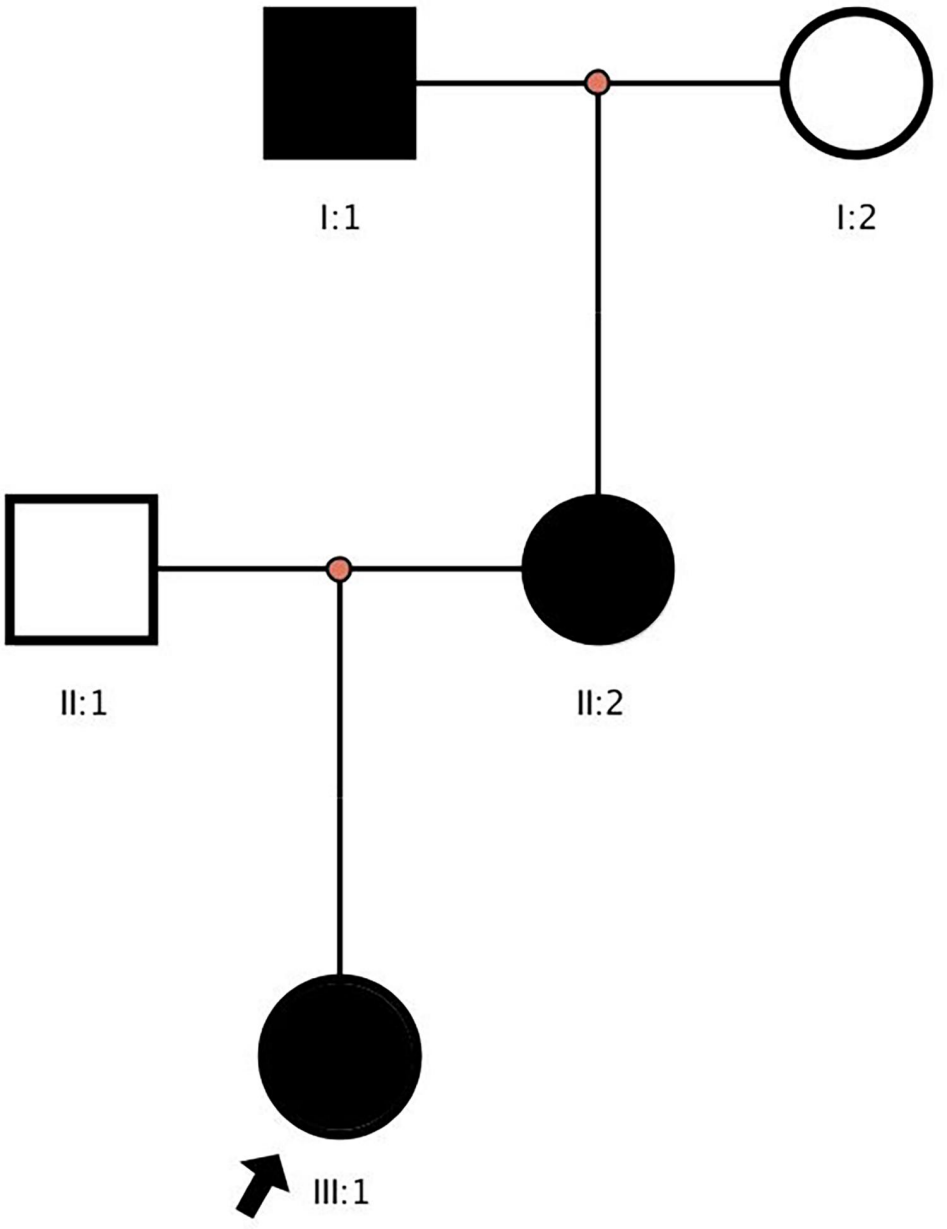
Family pedigree. Arrow marks the proband. Black: Family members with HypoPP syndrome.

Physical examination revealed that she had hypoplastic teeth and a high palatal arch (Figure 1A). The patient presented with hypoplastic nails on the second to fourth fingers of both hands (Figure 1B). All toenails, as well as the nails of the first and fifth digits, were absent (Figure 1C). Her hair, skin and oral mucosa showed no apparent abnormalities.

x-ray examination revealed dental hypoplasia (Figure 2). The distal phalanxes of the left thumb, index finger, and right thumb became pointed (Figure 2C). Segmented phalanxes were visible on the distal phalanxes of the middle finger, ring finger, and little finger of both hands (Figure 2C). No obvious abnormalities were found in the phalanges, metacarpal bones, and carpal bones of the remaining fingers. The distal phalanx of both feet became pointed (Figure 2B). The skeletal and joint structures appeared normal. The electrolyte testing indicated that the serum potassium level was 2.40 mmol/L. Other vital signs and parameters, including cardiac and renal function, thyroid function, sodium, chloride, calcium and magnesium levels, urinary electrolytes, 2-ketoglutarate, and plasma renin activity, were normal. Furthermore, no history of syphilis, hepatitis, trauma, drug, or food allergies was documented. The karyotype was 46, XX, indicating that the individual is female with no detectable abnormalities. WES revealed heterozygous variants in the *ATP6V1B2* and *CACNA1S* genes respectively, associated with the child's presentation. The *ATP6V1B2* variant was *de novo*, whereas the *CACNA1S* variant was maternally inherited (Figure 3).

Diagnosis: DDOD and HypoPP.

The management of the patient's dual diagnosis requires a multifaceted approach. For acute hypokalemic episodes, intravenous potassium chloride supplementation is administered under continuous cardiac monitoring to prevent life-threatening arrhythmias. For long-term prophylaxis, she is maintained on daily oral potassium supplements and is strictly advised to adhere to a diet low in sodium and carbohydrates to minimize attack triggers. Under this regimen, the frequency and severity of her paralytic attacks have been significantly reduced. The patient's mother and maternal grandfather also reduced the frequency of attacks by taking oral potassium supplements regularly.

Although there is no curative treatment for DDOD, a proactive supportive care plan has been implemented. This includes the use of cochlear implant for auditory rehabilitation and regular dental evaluations to manage enamel hypoplasia. Formal audiometric data were unavailable, but the patient now communicates normally with cochlear implants. At the latest follow-up, she attends school regularly and demonstrates normal communication and good quality of life. Dermatological consultations are scheduled to monitor her nail status and provide topical care to prevent periungual infections.

## 3 Discussion

The co-occurrence of DDOD and HypoPP in this patient presents a rare but diagnostically important scenario. While both conditions are well-described individually and caused by mutations in distinct genes (*ATP6V1B2* and *CACNA1S*, respectively), their simultaneous presence in a single individual is exceedingly rare and poses a diagnostic challenge.

The *ATP6V1B2* variant identified in our patient leads to a premature stop codon, resulting in loss-of-function of a critical subunit of the vacuolar H+-ATPase (V-ATPase) (1). This enzyme complex regulates lysosomal acidification and intracellular pH homeostasis, particularly within epithelial and neuroectodermal cells (1, 2). Mutations in *ATP6V1B2* are associated with the characteristic features of DDOD, including congenital sensorineural hearing loss and ectodermal defects such as hypoplastic nails and dental anomalies (1, 9).

In contrast, the *CACNA1S* variant (p.Arg528His) disrupts the voltage-gated calcium channel Cav1.1 in skeletal muscle, impairing excitation–contraction coupling and leading to episodic muscle weakness and hypokalemia characteristic of HypoPP (4, 5). This maternally inherited variant is a predominant pathogenic allele in familial HypoPP. Furthermore, the observed intrafamilial variability—with the mother being less severely affected than the grandfather—coupled with the amelioration of symptoms in both after age 40, is entirely consistent with the established natural history of HypoPP.

Although no direct pathophysiological interaction between V-ATPase dysfunction and calcium channelopathies has been established, both gene products play roles in ion homeostasis. Some studies have suggested that impaired acid–base regulation—such as that caused by V-ATPase dysfunction—may secondarily affect potassium handling or cellular excitability, particularly in renal and neuromuscular systems (10). For instance, renal tubular acidosis caused by defects in V-ATPase subunits has been linked to altered serum electrolyte profiles (11). Although *ATP6V1B2* and *CACNA1S* are involved in distinct biological systems—proton transport in lysosomes and calcium conductance in skeletal muscle—their coexistence may suggest a potential convergence in cellular ion homeostasis. The disruption of *ATP6V1B2* impairs vacuolar acidification and intracellular pH regulation, potentially altering the electrochemical gradient across membranes (12, 13). Similarly, *CACNA1S* mutations affect voltage-dependent calcium influx, indirectly influencing potassium handling and resting membrane potential (14). While current evidence does not support a direct molecular interaction, the co-occurrence of both defects could produce additive disturbances in cellular excitability and muscle function. This highlights the complexity of multilocus disorders and the need for functional studies exploring cross-pathway ion dynamics.

Previous cases have documented periodic paralysis with hearing impairment. A report described a woman with familial periodic paralysis within a family affected by Waardenburg syndrome (15). And another case detailed a man with thyrotoxic HypoPP who experienced sudden deafness (16). With the increasing adoption of broad-genome and exome sequencing in rare disease cohorts, the phenomenon of dual or multiple molecular diagnoses is becoming well-recognized. Studies have shown that up to ∼4%–5% of individuals with a suspected Mendelian disorder may harbour pathogenic variants in more than one gene locus, resulting in blended or overlapping phenotypes (6, 7). In a recent series of five pediatric patients with dual rare genetic diseases, researchers emphasized the importance of considering multiple genetic causes when clinical presentations are atypical or extend beyond a single syndrome (17). However, no published reports have directly implicated *ATP6V1B2* mutations in the pathogenesis of periodic paralysis or potassium imbalance. Therefore, in the absence of direct molecular or physiological overlap between these two pathways, we interpret this case as a rare but coincidental co-occurrence of two Mendelian disorders. Our case not only extends the phenotype of *ATP6V1B2* and *CACNA1S*-related disorders but also exemplifies the diagnostic challenge posed by multilocus genetic overlap.

This case exemplifies how integrative clinical evaluation combined with modern genomic technologies can uncover complex multilocus etiologies underlying atypical phenotypes. Compared with previous DDOD or HypoPP cases reported separately, our patient exhibited combined features but no additional systemic involvement, supporting the concept of blended phenotypes arising from multilocus variation. The diagnosis of this patient was achieved through comprehensive clinical assessment and trio-based WES, which identified two pathogenic variants in *ATP6V1B2* and *CACNA1S*. Nevertheless, the combined clinical phenotype could easily mislead diagnostic interpretation, particularly if only one aspect (e.g., hearing loss) is pursued. Such genome-wide approaches are increasingly recommended for patients with atypical or blended phenotypes because they enable the detection of multilocus pathogenic variants that would otherwise remain undiagnosed (6, 18).

The mainstay of therapy for HypoPP involves oral or intravenous potassium supplementation during attacks and long-term prophylactic administration of potassium salts to maintain normal serum potassium levels. Trigger avoidance—particularly of strenuous exercise, high-carbohydrate meals, or fasting—is crucial for reducing episode frequency. Carbonic anhydrase inhibitors such as acetazolamide and dichlorphenamide can be effective in selected patients by stabilizing membrane excitability (4, 5). For DDOD syndrome, no disease-specific treatment is currently available, and management focuses on auditory rehabilitation via cochlear implantation and supportive care for ectodermal manifestations.

Future therapeutic directions include gene-targeted therapies and channel-modulating compounds aimed at correcting ion-homeostasis defects at the molecular level. Advances in CRISPR-based genome editing and other precision genome-engineering tools have accelerated the translational outlook for correcting monogenic defects in human disease (19). In parallel, antisense oligonucleotide (ASO) and other nucleic-acid–based approaches are increasingly being developed as personalized therapies to modulate expression or splicing of disease-causing transcripts, and have shown proof-of-concept in several rare genetic disorders (20, 21). Functional investigations using induced pluripotent stem cell (iPSC)-derived tissues could elucidate whether combined lysosomal and calcium-channel dysfunctions produce additive or compensatory effects. Although translation of these modalities to skeletal-muscle channelopathies (such as *CACNA1S*-related HypoPP) or *ATP6V1B2*-linked disorders will require tissue-targeted delivery and careful safety evaluation, these technologies represent promising avenues for future personalized interventions.

Overall, this dual-diagnosis case highlights the clinical importance of integrating genomic diagnostics into patient management and foreshadows a precision medicine era in which therapy is tailored to individual molecular defects. Compared with previous DDOD or HypoPP cases reported separately, our patient exhibited combined features but no additional systemic involvement, supporting the concept of blended phenotypes arising from multilocus variation. These advancements collectively underline the future potential of genomic medicine for such multilocus conditions.

## 4 Conclusion

This report describes the first documented coexistence of DDOD and HypoPP in a single patient, expanding the clinical spectrum associated with *ATP6V1B2* and *CACNA1S* variants. The findings emphasize the diagnostic and clinical value of broad genetic testing in patients presenting with overlapping or unexplained phenotypes. Although current evidence supports coincidental co-occurrence rather than a shared pathogenic mechanism, potential interactions in ionic and metabolic pathways warrant further investigation. This case also underscores the emerging paradigm of precision medicine, in which comprehensive genomic analysis informs diagnosis, management, and future therapeutic strategies for rare genetic diseases.

## Data Availability

The original contributions presented in the study are publicly available. This data can be found here: https://apply.hgrg.net/login/2026BAT00058.
